# Feasibility and early cosmetic outcome of modified lateral intercostal artery perforator flap after breast conservative surgery

**DOI:** 10.1186/s12893-024-02367-6

**Published:** 2024-03-02

**Authors:** Islam M. Korayem, Rabie Ramadan, Haytham Fayed

**Affiliations:** 1 Department of Surgery, Faculty of Medicine, Alexandria University, Alexandria, Egypt; 2 Department of Surgery, Medical Research Institute, Alexandria University, Alexandria, Egypt

**Keywords:** Modified lateral intercostal artery flap, Breast-conserving surgery, Breast reconstruction, Lumpectomy, Cosmetic outcome

## Abstract

**Background:**

The lateral intercostal artery perforator (LICAP) flap aims at replacing the excised breast lump with axillary tissue rich blood supply. The purpose of this study is to report the initial results of a modification LICAP flap technique in terms of intraoperative technical feasibility and short-term cosmetic outcomes in the early postoperative period.

**Methods:**

Modified LICAP flap technique was performed on 36 female patients with pathologically proven BC located in the outer half of the breast in the period from June 1, 2021, to April 30, 2022.

**Results:**

The LICAP flap modification enabled performing the procedure with the patient in supine position without repositioning. The majority of the patients (90%) had satisfactory early cosmetic results as reported by the patients and oncoplastic independent surgeon.

**Conclusion:**

Modified LICAP flap reconstruction is feasible to be performed with the patient in supine position without repositioning and it has satisfactory early cosmetic outcomes.

## Introduction

Over the past few decades, breast-conserving therapy (BCT) has evolved and became the standard of care for patients with small, localized, and early breast cancer (BC) [1–3]. BCT entails breast conserving surgery (BCS) in the form of lumpectomy followed by breast radiotherapy to eradicate microscopic residual disease left in the breast [4]. 

Despite the adequate oncologic outcomes after BCS became well established, their relatively limited indications and mediocre aesthetic outcomes remain challenging as patients continue to express cosmetic dissatisfaction owing breast asymmetry due to reduced breast volume as well as breast contour deformities [5]. 

This represented the pinnacle for oncoplastic breast-conserving surgery (OBCS) which emerged to bridge the gap between conventional BCS and mastectomy. Oncoplastic BCS helped to expand the extent of tumors that could be managed by BCS and allowed resection of larger breast tumors with adequate resection margins while maintaining optimal functional and cosmetic outcomes through volume displacement and volume replacement [6]. 

Volume displacement involves parenchymal reshaping using the remaining breast tissue to fill the defect after lumpectomy. Alternatively, volume replacement entails breast reconstruction by adding extra volume to compensate for lumpectomy defect by utilizing loco-regional flaps from outside the breast (e.g., the axillary region or upper abdomen) [6]. 

Nonetheless, volume displacement techniques were associated with potentially reduced ipsilateral breast resulting in a noticeable breast size discrepancy with the consequent need for contralateral breast reduction to maintain symmetry. This limitation is not, however, met with volume replacement techniques which help maintain breast symmetry and omit the need for further contralateral breast procedure giving it a clear advantage over breast displacement procedures [6, 7]. 

The techniques for breast volume replacement have progressed from bulky musculocutaneous and fascio-cutaneous flaps to more complex perforator tissue flaps which utilize only skin and subcutaneous tissue for reconstruction of breast defects while preserving fascia, muscles and nerves which are left in their native place to serve their original function and minimize donor site morbidity [8, 9]. 

Hamdi et al. pioneered several highly versatile flaps for use in breast reconstruction. His lateral intercostal anterior perforator flap was described as a flap based on the perforating arteries which originate in the costal segment of the intercostal arteries [8]. The most utilized pedicled perforator flaps for breast reconstruction include the thoracodorsal artery perforator (TDAP) flap, the intercostal artery perforator (ICAP) flap, and the serratus anterior artery perforator (SAAP) flap [8, 10]. 

The lateral intercostal artery perforator (LICAP) flap offers the advantages of repurposing excess local axillary tissue while maintaining a reliable blood supply. These flaps help fill the remnant cavity to minimize subsequent defects. It is reported that the surgical procedure is not time-consuming and has minimal morbidity as the flap is harvested without jeopardizing the muscles or nerves at the donor site [8, 11]. However, such procedure faced 2 limitations; the patient needs intraoperative repositioning from supine to lateral position to allow harvesting the flap following lumpectomy. Additionally, the resultant scar was quite visible as it extended by approximately 5 cm from the lateral mammary sulcus to the posterior axillary line [11–13]. To avoid those challenges, Meybodi et al. [14] proposed a modification through making a lazy S incision along the inferior and lateral mammary fold which extended in the lower axilla allowing better axillary access without patient repositioning as well as making the scar less visible. [14]

We sought to report our initial experience with further refinement of the modified LICAP technique to assess its feasibility and short-term cosmetic outcomes related to the surgical modification within the early postoperative period.

## Patients and methods

The study was performed in 2 medical centers that are directly affiliated to Alexandria University: Surgical Oncology Unit of the Department of Surgery in Alexandria University Main Hospital, and the Department of Surgery in the Medical Research Institute. We included female patients presenting with pathologically proven BC located in the outer quadrants of the breast which fulfilled the criteria for BCS. An informed consent document was obtained from all patients who agreed to participate in this study after thorough explanation of the procedure. The study was approved by the Ethics Committee and the Institutional Review Board of Alexandria Faculty of Medicine (IRB No. 00012098 – FWA No. 00018699) and the Medical Research Institute under serial number 0305610.

### Surgical technique

**Fig. 1:**
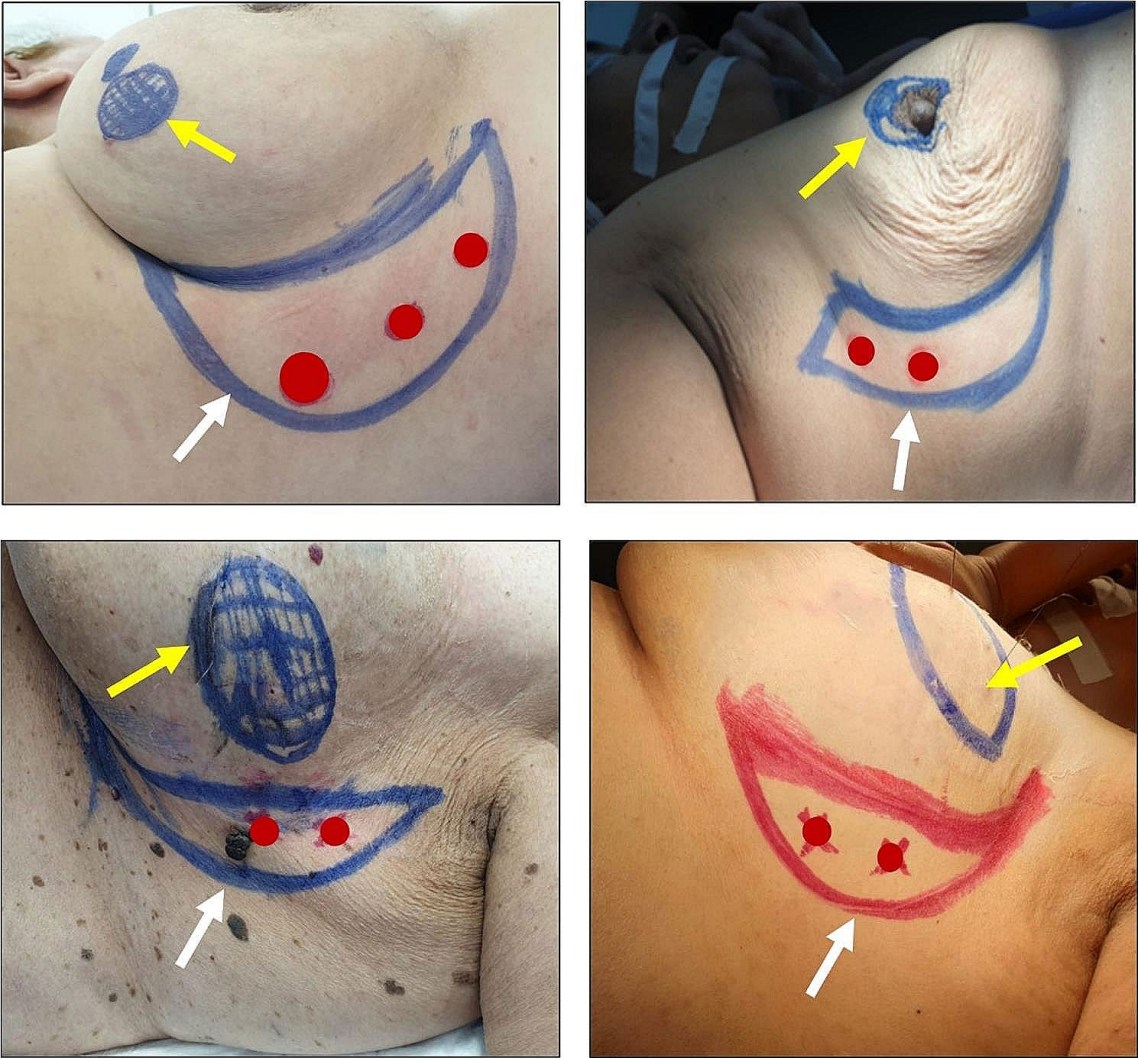
Examples of marking of the primary tumor-site in the breast (yellow arrows), delineating the sickle-shaped flap (white arrows) bounded by 2 semilunar lines and enclosing the LICA perforators (red dots)

The patient was placed in supine position with the ipsilateral arm abducted at 90 degrees. The location of the breast tumor was initially determined and marked. A handheld doppler probe was used to accurately localize and mark the perforator branches of the lateral intercostal artery. We used to mark the perforators that were the closest to the breast lesion to avoid excessive width of the flap. The flap was then designed by drawing an initial semilunar line in the lateral mammary sulcus which represented the medial margin of the flap. It could be extended downwards to reach the inframammary fold based on the location of the tumor within the breast. A second semilunar line was drawn inferior and lateral to the initial one and at a distance almost equivalent to the width of the expected lumpectomy defect and represented the lateral margin of the flap. The final shape of the flap looked like a sickle and enclosed the previously marked perforators (Fig. 1). We made sure the width of the flap was not excessively large and that the donor site had abundant remnant tissue to enable approximation and direct closure of the wound edges at no tension and to prevent any vascular compromise.

**Fig. 2:**
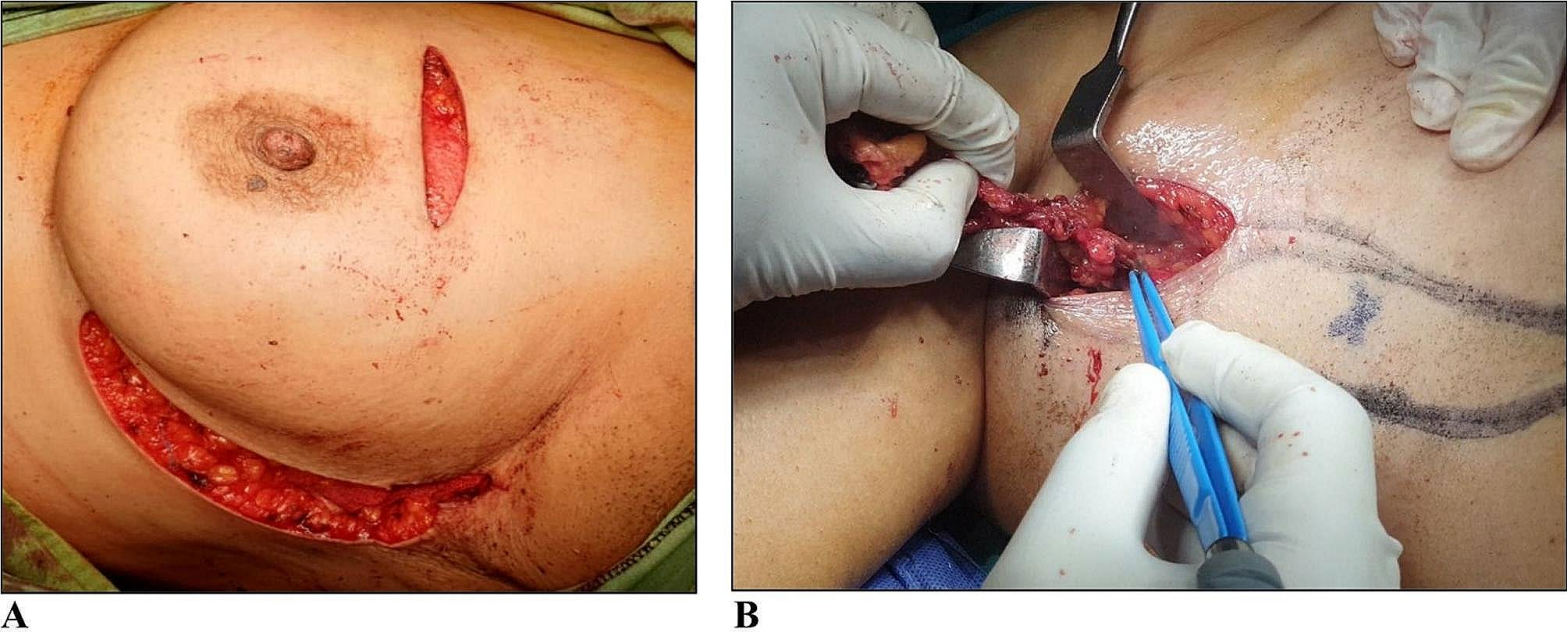
**(A)** Lumpectomy performed through a separate incision for being attached to the overlying skin. **(B)** Axillary dissection is performed through the same incision of the flap prior to flap harvesting

**Fig. 3:**
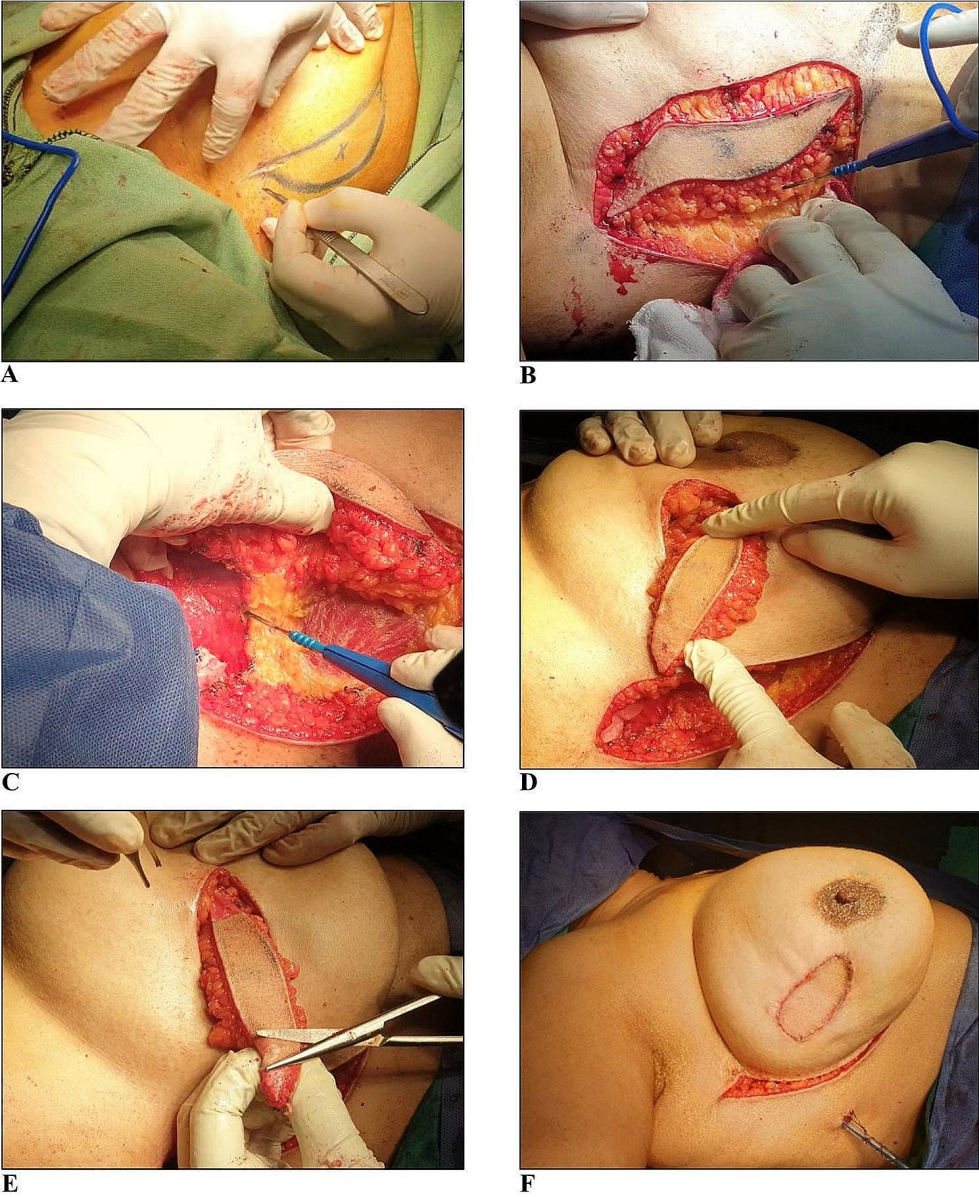
Steps of flap harvesting which begins with incision and dissection of the flap according to the previously drawn margins (**A** & **B**), harvested flap shape and thickness after completion of dissection until the mapped perforator is reached **(C)**, flap is then passed through a tunnel made in the SC tissue and flipped with gentle manipulation to fill the lumpectomy defect while not jeopardizing the vascular supply **(D)**, de-epithelialization of excess skin from flap edges to fit in the defect **(E)**, flap is fixed in the lumpectomy defect by sutures followed by direct closure of donor site **(F)**

The procedure began with wide local excision of the breast lump through the planned flap incision. A separate lumpectomy incision was resorted to whenever the breast mass was attached to the overlying skin (Fig. 2A) or retro-areolar in location. Intraoperative frozen section was routinely performed to confirm negativity of all the resection margins all around and deep to the tumor. Sentinel lymph node biopsy (SLNB) and/or axillary lymph node dissection (ALND) was performed through the lateral edge of the flap incision prior to the flap-harvesting procedure (Fig. 2B). Once negativity of the resection margins was confirmed, our attention was shifted towards harvesting the flap proceeding from lateral to medial edge (Fig. 3A and B) while carefully identifying and preserving the previously mapped perforators keeping them within abundant mesentery of fat (Fig. 3C) to enable free flap mobilization to reach its final destination and fill the lumpectomy defect without significant vascular compromise (Fig. 3D). Minimal flipping, rotation, or folding may be required to accommodate the flap in the lumpectomy defect whenever there was discrepancy between the defect size and the flap size as seen in patients with normal body mass index (BMI) who lack abundant axillary tissue or those with small breast size. In cases where the tumor was excised from the same flap incision without extra skin defects, the flap was completely de-epithelialized to position it subcutaneously in the lumpectomy defect followed by direct closure of the lone donor site defect. If lumpectomy was performed through a separate skin incision with resultant skin defect, the dimensions of the flap were tailored to keep a skin island equivalent to the lumpectomy defect in one of its dimensions with de-epithelialization of any excess skin from the edges as shown in Fig. 3E. The flap was temporarily held in position using nonabsorbable stay sutures followed by fixation to the edges of the defect using absorbable deep subdermal and subcuticular sutures after placement of a negative-suction drain in the lumpectomy site and axilla, if ALND was performed. The wound edges of the flap donor site were approximated and directly closed using absorbable sutures without a drain (Fig. 3F). We used to tuck the medial edge of the flap donor site to the underlying muscle to fix it in position and limit its mobility while undermining the lateral edge of the wound whenever further tissue liberation is required to make the lateral wound edge move freely towards the medial wound edge to allow for sound closure of the donor site defect at no tension and prevent any form of lateral deviation of the breast. Additionally, this added better cosmesis by making the wound stay more medially and hide in the lateral mammary sulcus behind the breast. In all cases, marking clips were routinely placed in the lumpectomy cavity prior to mobilization and fixation of the flap.

Patients were discharged the next day and scheduled for routine outpatient follow up at 1, 2, 4, 8, and 12 weeks postoperative to assess for any surgical complication and early cosmetic outcome. More frequent visits were required whenever surgical complications were encountered to allow for better care and thorough follow-up.

### Data collection

Patients’ demographic data, preoperative clinical and imaging data, intraoperative details for the breast and axilla, time for frozen section, total operative time (from first skin incision till last skin stitch), tumor histopathologic details, postoperative morbidity and mortality, and cosmetic results were recorded. Early cosmetic results were assessed by the patients, as well as an independent surgeon, within the first 12 weeks-postoperative using the Harvard Scale (four-point Likert Scale) according to the following definitions: [15, 16]

- Excellent: the treated breast is almost identical to the untreated one.
- Good: the treated breast is slightly different from the untreated one.
- Fair: the treated breast is clearly different from the untreated one, but it is not seriously distorted.
- Poor: the treated breast is seriously distorted.

### Statistical analysis

Descriptive statistics were reported for the whole cohort. Continuous variables were represented as means, standard deviation (SD), and ranges, whereas categorical variables were represented as numbers and percentages. All the analyses were performed using IBM® SPSS® Statistics version 26 (IBM Corporation, Armonk, NY).

## Results

Thirty-six female patients with BC were included in this study and underwent our modified LICAP technique in the period from June 1, 2021, to April 30, 2022. Table 1 summarizes patient’s demographic data and associated comorbidities.

**Table 1:** Patients demographic data and associated comorbidities

Variable	
**Age (years)**	48 ± 8.9, (29–65)
≤ 50	20 (55.6%)
> 50	16 (44.4%)
**Marital status**	
Single	1 (2.8%)
Married	35 (97.2%)
**BMI (kg/m** ^**2**^ **)**	26.9 ± 2.2
Normal (18.5 - <25)	3 (8.3%)
Overweight (≥ 25)	33 (91.7%)
**Use of OCPs**	10 (27.8%)
**Positive family history for BC**	6 (16.7%)
**Associated comorbidities**	15 (41.7%)
DM	13 (36.1%)
HTN	6 (16.7%)
**Breast size (cup)**	
A	6 (16.7%)
B	21 (58.3%)
C	9 (25.0%)

*Continuous data are reported as mean ± SD, (range)*

*Categorical data are reported as n (%)*

### Patient presentation and tumor characteristics

All the patients presented with painless breast lump in the absence of any reported nipple discharge or skin changes. Breast lump was found in the left breast among 23 patients (64%) and in the right breast among 13 patients (36%). The majority of the patients had their lump in the outer half of the breast (*n* = 28, 78%), 22 patients (61%) in the upper outer quadrant (UOQ) with 3 of them (8%) being bifocal, and 6 patients (17%) in the lower outer quadrant (LOQ). The remaining 8 patients (22%) had supra-areolar breast lump. Axillary lymph nodes were clinically palpable in 10 patients (28%).

Preoperative mammography identified the breast lump among all patients with average size of 2.86 ± 0.86 cm in the in the largest dimension (range: 1–5 cm). Microcalcification was found in 4 patients (11%) and suspicious axillary lymph nodes were detected in 12 patients (33%), 10 of which were detected on clinical basis. Neoadjuvant chemotherapy was given to 8 patients (22%).

### Intraoperative details

All the 12 patients (33%) clinically or radiologically proven suspicious axillary lymph nodes were managed by axillary lymph node dissection and all of them had metastatic deposits. The remaining 24 patients with negative axilla for suspicious lymph nodes by clinical examination or mammography were subjected to SLNB among which, 6 (17%) had metastatic deposits on frozen section which mandated completion with axillary clearance.

The majority of our patients had the whole surgery performed through the same flap incision (*n* = 25, 69.4%). Eleven patients (30.6%) required a separate lumpectomy incision for having the mass retro-areolar in location in eight patients (22.2%) where lumpectomy was achieved through a separate circum-areolar incision (modified round block technique). The remaining three patients (8.3%) had their breast mass attached to the skin which required excision of the mass with the overlying skin in toto to ensure adequate safety margin.

### Postoperative outcomes

All of the 36 patients were found to have invasive ductal carcinoma as documented by the final postoperative histopathological report. None of the 8 patients who received neoadjuvant chemotherapy sustained a complete pathological response.

Results of postoperative surgical and cosmetic outcomes are outlined in Table 2. No postoperative mortality was recorded among our patients. Nine patients (25%) developed postoperative wound complications which were managed conservatively in all of them. The most common morbidity was seroma formation (*n* = 6, 16.7%) which was managed by serial needle aspiration under local anesthesia and under complete aseptic technique in the outpatient clinic until no further fluid was identified on ultrasound basis. Two patients (5.6%) experienced wound dehiscence and superficial wound infection which was managed by repeated daily dressing with coverage by oral and topical antibiotics. The remaining patient (2.8%) developed a small hematoma in the wound which was managed by warm fomentation and topical creams under umbrella of antibiotics until it resolved within 11 days. None of the patients experienced flap/fat necrosis.

**Table 2:** Postoperative histopathologic data, morbidity, and cosmetic outcomes

Postoperative outcomes	No. (%)
**Pathological data**	
**Gross weight of the specimen (mg)**	63 ± 13.1, (39–100)
**Tumor grade**	
I	2 (5.6%)
II	27 (75%)
III	7 (19.4%)
**Biological subtype**	
Luminal A	29 (80.6%)
Luminal B	1 (2.8%)
Her-2 enriched	2 (5.6%)
Triple-negative	4 (11.1%)
**Operative time (min)**	
Time for frozen section	48.5 ± 11.9, (30–90)
Overall operative time	128.9 ± 42.1, (90–260)
**Postoperative complications**	
Seroma	6 (16.7%)
Wound dehiscence	2 (5.6%)
Hematoma	1 (2.8%)
**Cosmetic outcomes**	
**Independent Surgeon assessment**	
Excellent	24 (66.7%)
Good	8 (22.2%)
Fair	4 (11.1%)
**Patient satisfaction**	
Excellent	30 (83.3%)
Good	2 (5.6%)
Fair	4 (11.1%)

*Continuous variables are reported as mean ± SD, (range)*

*Categorical data are reported as n (%)*

Thirty patients (83.3%) reported excellent cosmetic outcome based on their survey, and 2 (5.6%) reported good outcomes. From the independent surgeon’s assessment, excellent cosmetic outcomes were encountered in 24 patients (66.7%), and good outcomes in 8 patients (22.2%). Six patients were shifted from the excellent outcome to good outcome by the independent surgeon due to the presence of slight change of nipple level and/or deviation among 4 patients, and the presence of wide / hypertrophic scar due to superficial wound infection and dehiscence which healed later by secondary intention. There were no poor cosmetic results reported by either the patients or the assigned independent surgeon.

**Fig. 4:**
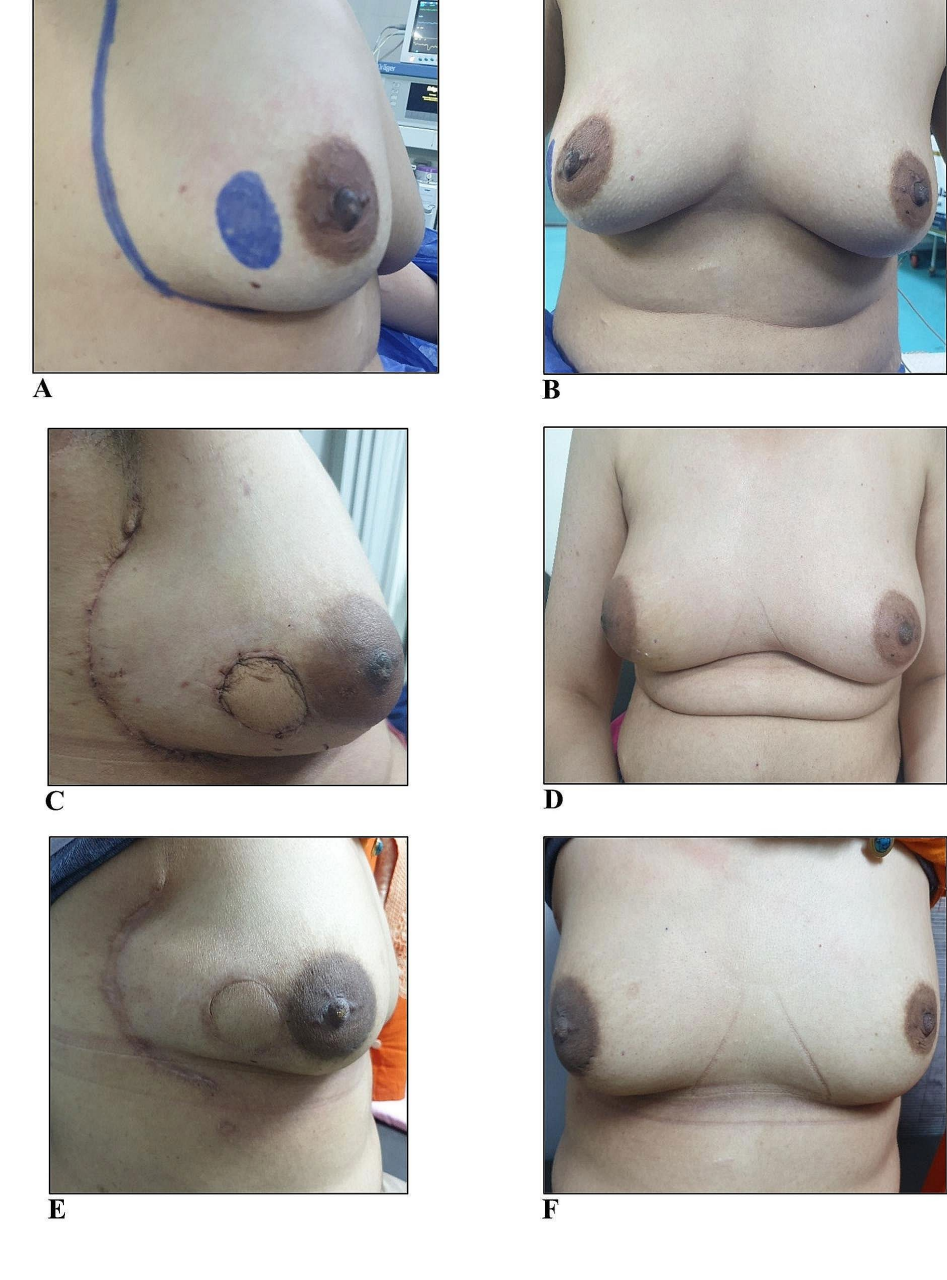
Preoperative lateral and frontal views showing the marking of right breast lump and the medial margin of the planned LICAP flap (**A** & **B**), 2-week-postoperative lateral and frontal views showing scars of the flap donor site and the flap filling a separate lumpectomy site (**C** & **D**), delayed postoperative lateral and frontal views of the patient 6 months after radiotherapy (**E** & **F**)

Figure 4 shows lateral and frontal views of the breast for a patient who had right breast outer quadrant mass in three phases; preoperatively (Fig. 4A and B), 2 weeks postoperative (Fig. 4C and D), and 6 months post-radiotherapy (Fig. 4E and F). She received BCS where the lumpectomy was performed through a separate incision leaving a defect in the breast with immediate reconstruction using the modified LICAP flap to substitute the lumpectomy volume defect and bridge the skin defect created by lumpectomy with acceptable cosmetic outcomes as shown at 2-week-postoperative and 6 months after radiotherapy.

**Fig. 5:**
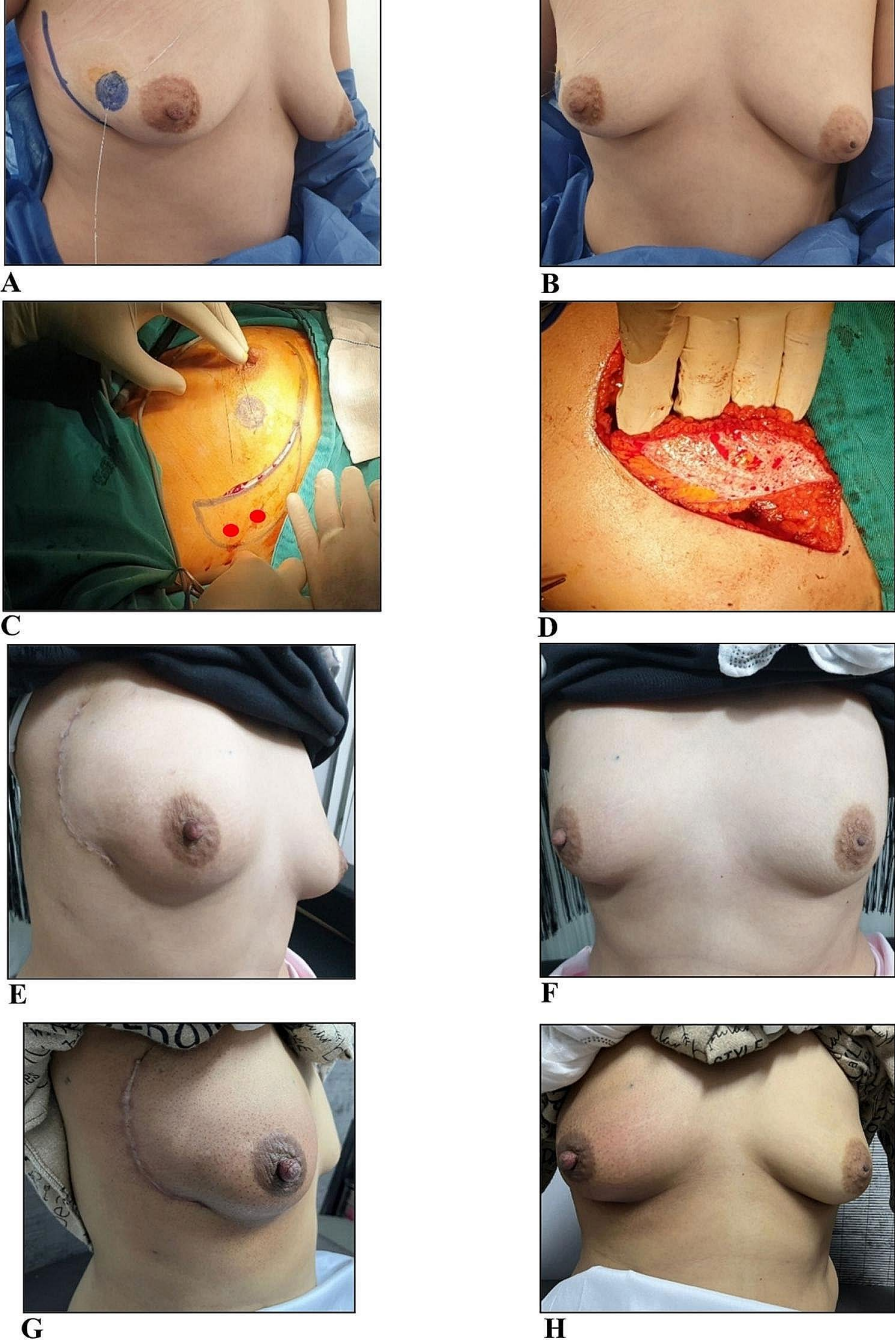
Preoperative lateral and frontal views of a female patient with normal BMI and small breast (cup size **A**) sitting on the operating table with her arms adducted to her side and showing the marking of the right breast lump as well as delineation of the medial margin of the planned LICAP flap (**A** & **B**), intraoperative images showing the modified LICAP flap before and after harvesting with the mapped perforators (red dots) which required de-epithelialization to fill the lumpectomy defect after excision of the mass through the same wound of the flap (**C** & **D**). Postoperative cosmetic outcomes at 1-month-postoperative (**E** & **F**) are seen through lateral and frontal images of the breast while patient abducting her arms > 90 degrees to expose the scar with good healing. Cosmetic outcomes after 4 months of surgery and 1 month of radiotherapy with resultant post-radiotherapy mild right breast edema and inflammation (**G** & **H**)

Figure 5 depicts a female patient with normal BMI and small breast size (cup size A) throughout the preoperative, intraoperative, and postoperative phases in a chronological order. Figure 5A and B display preoperative lateral and frontal views of the patient while sitting on the operating table with her arms adducted to her side making her breast slightly ptotic with marked right outer quadrant lump as well as delineated medial margin of the planned LICAP flap. Intraoperatively, Fig. 5C shows beginning incision of the flap wound after complete delineation of the lateral and medial margins of the LICAP flap making it look like a sickle and including the 2 red-marked perforators. Figure 5D shows the final form of the flap after harvesting and de-epithelialization making it ready to fill the lumpectomy defect which was made through the same wound of the flap with no separate skin incision for the lumpectomy. Figure 5E and F show lateral and frontal postoperative views of the breast during the patient’s visit at 1-month after surgery. It is apparent that the patient was positioned with her arms abducted by more than 90 degrees to better expose the scar, which expressed good healing, and to identify any sort of breast / nipple deviation, which was not appreciated. Figure 5G and H show the lateral and frontal views of the patient’s breast 4 months postoperatively and after 1 month of radiotherapy with good cosmetic outcomes regarding the flap donor-site scar and breast / nipple position apart from apparent mild radiotherapy-induced inflammation and edema as seen in the operated right breast.

## Discussion

Pedicled perforator flaps were described in partial breast reconstruction for defects not exceeding 30% of breast volume. In their initial experience, Hamdi et al. [8, 12, 13] published extensively on pedicled perforator flaps and described the original LICAP flap for partial breast reconstruction. They relied on anatomical landmarks to identify the perforators intraoperatively and patient repositioning was mandatory to shift from lumpectomy to flap harvesting since the flap incision was made quite away from the lumpectomy incision.

In our series, we used a handheld doppler to map the nearest perforators to the lesion and skin-marked them prior to skin incision. The entire procedure of lumpectomy, axillary clearance, and flap harvesting, was performed with the patient lying in the same supine position without the need for repositioning. This is similar to the maneuver of perforator mapping and patient positioning described by Meybodi et al. [14]

Two-staged approach where BCS is followed by interval breast reconstruction was described by Roy and Tenovici [17]. They described this approach specifically for patients with high tumor-to-breast ratio (> 30%) to guard against unnecessary mastectomy and to ensure tumor resection with adequate safety margins. The initial step entailed wide local excision of the tumor followed by filling the lumpectomy cavity with saline. The second stage of flap reconstruction ensued after 2–4 weeks only after retrieval of pathology reports documenting complete tumor excision with adequate safety margins [17]. In our experience, we adopted a single stage approach where lumpectomy and breast reconstruction were performed simultaneously. We did not require a two-stage approach since none of the patients we encountered had high-risk breast tumor with high tumor-to-breast ratio. Additionally, intraoperative frozen section was mandatory in all of our procedures to confirm adequate tumor safety margins prior to proceeding with flap-harvesting for reconstruction.

In terms of cosmetic outcomes of LICAP flap, several studies reported favorable outcomes. Lipman et al. [11] performed 16 LICAP procedures for breast augmentation among 12 patients who had previous breast surgery or massive weight loss. They reported satisfactory outcomes among all of the patients with no complaints regarding the flap scar. Similarly, Kim et al. [18] reported satisfactory flap cosmetic outcomes (excellent and good) after using the flap for reconstruction in 40 patients. Their method for assessment of cosmesis included surveys conducted by the surgeons as well as the patients themselves.

In our experience, we used a survey to assess cosmesis conducted by patients and an independent surgeon. Satisfactory cosmetic outcomes (excellent and good) were reported by majority of the patients (*n* = 32, 88.9%). Several factors played a key role in attaining such favorable cosmetic outcome observed. We suppose that the sickle shape of the flap that we modified and its placement in the lateral mammary sulcus have helped to keep the scar hidden behind the breast without visible extension in the axilla. Additionally, it was interesting to observe that patients who presented with tumors in the outer quadrants of the breast and deeply seated in the breast with no fixation to the overlying skin had the best cosmetic outcome since they had the advantage of performing the entire procedure through a single wound (i.e. the LICAP flap wound) with no separate lumpectomy incision required hence leaving a single scar that is hidden in the sulcus. Patients with retro-areolar breast cancer came next in terms of satisfactory cosmetic outcomes as the lumpectomy incision was also relatively hidden.

It is important to mention that most of our patients were overweight (91.7%) which has definitely predisposed to the presence of relative breast ptosis and abundant skin and subcutaneous tissue in the axilla. This has potentially allowed for adequate wound closure at minimal or no tension resulting in no significant breast traction or deviation to the ipsilateral side. While in our experience we performed modified LICAP flap among only 3 patients with normal BMI, we did not experience significant breast deviation or asymmetry. However, this finding should be taken cautiously and results from 3patients cannot be generalized on all thin patients with normal BMI. It is valid to assume that performing such procedure in thin patients with normal BMI would predispose to significant breast deviation and asymmetry upon closure of the flap wound. This could be explained by the higher degree of traction of the flap wound on the breast due to lack of abundant skin and subcutaneous fatty tissue in the axilla in those patients. This could also be encountered among patients in whom the most suitable perforator was found at a distance and away from the anterior border of latissimus dorsi muscle which will eventually result in a flap with relatively large width creating a big defect in the axilla.

Our study details a surgical technique of a proposed modification of LICAP flap. We report the results of the initial experience with this technique with the primary intention of assessing intraoperative feasibility of flap harvesting without patient repositioning, as well as evaluation of the early postoperative cosmetic outcome. The limitations of this study may be the relatively small number of patients included and the short postoperative follow-up period. It is recommended to perform this procedure on larger scale of patients with longer follow-up periods to better assess the cosmetic as well as oncologic outcomes of the procedure. Additionally, we will need to include more patients with normal BMI to be able to assess cosmetic outcome of the modified flap in such group of patients. A comparative study may be required to compare this technique to one or more of the modifications reported in literature for better illustration of the results on statistical basis.

## Conclusion

The Modified LICAP flap reconstruction made it technically feasible to perform the whole procedure with the patient in supine position without repositioning. Early cosmetic outcomes were best encountered among females with laterally located breast cancer not fixed to overlying skin as the entire procedure of lumpectomy and ALND was performed through a single and same wound used for harvesting of the LICAP flap leaving a single scar hidden in the lateral mammary sulcus. Patients with retro-areolar breast cancer still had satisfactory cosmetic outcomes as the two incisions for lumpectomy and flap were relatively hidden.

## Data Availability

We, the authors, confirm that the data and materials of this study are available and will be presented upon request.
